# The interplay between voluntary food intake, dietary carbohydrate-lipid ratio and nutrient metabolism in an amphibian, (*Xenopus laevis*)

**DOI:** 10.1371/journal.pone.0208445

**Published:** 2018-12-07

**Authors:** Andrea Brenes-Soto, Ellen S. Dierenfeld, Geert P. J. Janssens

**Affiliations:** 1 Laboratory of Animal Nutrition, Faculty of Veterinary Medicine, Ghent University, Merelbeke, Belgium; 2 Animal Science Department, University of Costa Rica, Ciudad Universitaria Rodrigo Facio, San José, Costa Rica; 3 Ellen S. Dierenfeld, LLC, St. Louis, Missouri, United States of America; University of Illinois, UNITED STATES

## Abstract

Digestion of food and metabolism of frogs are little studied at the moment, and such processes could be very particular in the case of amphibians, given their ectothermic and carnivorous nature which may lead them to use nutrients through specific biochemical pathways. In the present study, 24 adult *Xenopus laevis* (six replicates with two frogs per treatment) were randomly assigned to two diets with different carbohydrate:fat ratio (4.5:1 and 2.1:1), changing the dietary glucogenic and lipogenic proportions. Food intake (FI) per unit metabolic body weight (MBW) as well as macronutrient digestibility were calculated, and circulating blood acylcarnitines and amino acids measured, in order to evaluate the effect of the diet treatments. Results demonstrated that food intake regulated most of the changes in the parameters evaluated; significant differences were obtained in crude protein and fat digestibilities through the effect of FI/MBW (p<0.05), whereas diet treatment had a significant effect on the levels of malonyl-CoA. Food intake also significantly impacted malonyl, isovaleryl, hydroxyisovaleryl and long chain fatty acid concentrations; significant (p<0.05) interactions between those metabolites were detected owing to diet. The findings obtained suggest that food intake was the main factor controlling digestion and metabolism in *X*. *laevis*, with frogs preferring to utilise protein and fat as primary sources for energy production in the citric acid cycle, reflecting characteristics of a strict carnivore physiological model.

## Introduction

In nature, animals have to face diverse challenges to acquire the adequate profile of nutrients needed to assure health, reproduction and survival. Adaptation of a species to a determined habitat is defined by the presence of predators, shelter and the availability of food in terms of quantity and quality to cope with nutritional demands, dependent on the physiological stage of life [1]. In amphibians, diets and feeding habits are influenced by many constraints. Extrinsic factors include seasonal availability of food and abundance of competitors, while intrinsic factors involve ecological tolerances and morphological limitations related to ontogenetic stage, size and specialization. Adult anurans are principally carnivores, displaying different feeding strategies including the “sit and wait” or “jump” modes, which can, in turn, influence the energy expenditure of foraging activities. Consequently, diet composition must be appropriate, and utilisation efficient to meet the energetic costs of prey location, capture and ingestion, as well as the absorption of hydrolysed components after complete digestion [2,3].

The role of macronutrient availability or demand by organisms often varies widely over time and space, having large effects on how organisms interact with their environment across biological scales [4], but most animals do have the ability to adjust the percentage(s) of various macronutrients to fulfil their nutrient requirements [5]. The nutrition process encompasses several steps in the animal body: from ingestion—where the nutrients from the food are brought into the digestive system for physical, chemical and microbial breakdown during digestion, to absorption—during which the nutrients are transported to the different tissues, until their total metabolisation after a succession of chemical reactions leading to the synthesis or degradation of various compounds, and the subsequent excretion of waste substances [6]. As a first step, it has been demonstrated that voluntary food intake can be regulated by the dietary macronutrient profile in several carnivore species. For instance, dogs and cats have shown the ability to balance their macronutrient intake through selecting foods and combining them in required proportions, yielding a macronutrient (protein:fat:carbohydrate) ratio composition of 30:63:7% in the case of the more omnivorous dog [7], and 52:36:12% in the case of the obligate carnivorous cat [8]. Wolves (*Canis lupus*) presented a ratio of 54:45:1% [9] and a carnivorous fish, the rainbow trout (*Oncorhynchus mykiss*), 63.8:18.5:17.7% [5], all expressed in terms of the energy obtained from each macronutrient. Such relationships have not yet been studied in anurans. Although there are studies regarding feeding rate effects in terms of growth, weight gain, feed intake and feed conversion in the bullfrog *Rana* (*Lithobates*) *catesbeiana* [10,11], as well as the improvement of food intake after eating diets with high fat content in the false tomato frog *Dyscophus guineti* [12], the aptitude to self-regulate macronutrient uptake remains to be investigated.

In most amphibians, after ingestion the food is partially fragmented in the mouth, which allows the entry of the enzymes into the soft tissue of the prey, starting the digestion process [2], although physical breakdown mechanisms are widely variable and also depend on the prey exoskeleton characteristics [13]. In principle, the first step in nutrient utilisation is digestibility. It is assumed that the proportion of nutrients not excreted in the faeces were absorbed and utilised by the animal according to its requirements. Nonetheless, it is acknowledged that part of the faecal material is contributed by basal endogenous losses, enzymes, other substances secreted into the gut and not reabsorbed, as well as cellular material from the intestinal epithelium and other tissues, hence resulting values are termed “apparent” digestibility coefficients [6,14], with apparent digestibility calculated as (100-indigestible components). In anurans, apparent digestibility coefficients for dry matter, protein, fat, energy and amino acids have been obtained for the cultivated bull frog *R*. *catesbeiana* [15,16,17]. However, those studies are focused on productive performance of growing animals comparing different foodstuffs, rather than the biochemical pathways underlying those processes. The macronutrient ratio plays a determinant role in the overall utilisation of nutrients for their adequate function in the target tissues/cells. Within carnivores for instance, it has been reported that the mink (*Mustela vison*) preferred diets containing 35% protein and 50% fat [18], while studies with the hybrid fish aspikutum (*Leuciscus aspius* X *Rutilus frisii*) demonstrated that diets containing 35% protein and 15% fat were appropriate for optimum growth and feed utilisation [19]. Furthermore, the shi drum fish (*Urbina cirrosa* L.) also showed an enhancement in immune status eating diets containing 47% protein and 10% fat [20]. Macronutrient requirements and optimal protein:lipid or carbohydrate:lipid ratios have been poorly investigated in anuran amphibians, comprising few studies on cultivated species as such as the bull frog (carbohydrate:fat ratio 1.2–12.1:1) [17,21,22], the Thai native frog *Rana rugulosa* (protein:fat ratio 2.7:1) [23] and *Rana perezi* (protein:fat ratio 4:1) [24]. Response(s) in nutrient metabolism related to these dietary macronutrient profiles has not been evaluated to date in frogs.

The main transformation of nutrients in energy metabolism occurs through oxidation, following digestion, of food constituents to acetyl-CoA, the elemental substance for fat synthesis and energy production via ATP synthesis in the citric acid cycle [6]. Both pathways are interconnected to all nutrients through acetyl-CoA, but lipolysis can take place only when there is a surplus of available acetyl-CoA in the metabolic pool [25]. Fatty acids can be converted to CoA esters in the cytosol, being moved in and out of the mitochondria by transferring them from CoA to carnitine, which in the form of acyl carnitine can penetrate the inner mitochondrial membrane and functions as a modulator of intra- and extra- mitochondrial acyl-CoA compounds [26,27]. The major mechanism controlling the synthesis of fatty acids from glucose involves the carboxylation of acetyl-CoA to form malonyl-CoA, determining the rate of fatty acid oxidation. When glucose is actively converted to fatty acids, the concentration of malonyl-CoA is elevated, thus decreasing the transfer of fatty acyl residues from CoA to carnitine (and its translocation into mitochondria), depressing ß-oxidation; these processes substantially depend on the feeding status of the animal [26,28].

In general, a limitation in gluconeogenic substrates such as glucose, amino acids and propionate (from intestinal fermentation) leads to shortage of oxaloacetate, consequently inhibiting acetyl-CoA from entering the citric acid cycle [29]; this situation is known to exert a less efficient energy metabolism through ketone body synthesis, and will at a further stage lead to hepatic lipidosis (fatty liver disease) [30,31]. The extent to which particular gluconeogenic substrates can be used differs substantially among species, even among carnivores. For example, because wolves have long periods of non-feeding in nature, they possess amino acid sparing strategies in their metabolism, whereas frequent feeders such as cats maintain a high rate of amino acid use for energy purposes [9]. To date, it is not known whether amphibians fit into one or other of these physiological categories.

Given the carnivorous nature of adult anurans, viable comparisons may be made with other strict carnivores in terms of potential channels to provide energy, where lipogenic and proteinogenic pathways predominate over carbohydrate-based pathways. For example, in the domestic cat, a strict terrestrial carnivore, the iso-energetic substitution of fat, protein and starch revealed that protein had the strongest reducing effect on insulin sensitivity; this confirms the prominent role of protein compared to carbohydrates as gluconeogenic substrate in carnivore metabolism [32]. Likewise, in carnivorous fish, the efficiency of macronutrient utilisation for energy is also documented: there is increasing evidence that gluconeogenesis, glycolysis and ß-oxidation play different roles in energy provision compared to other vertebrates, likely related to a limited capacity for starch digestion [33], as reported for the barramundi (*Lates calcarifer*) [34] and the Atlantic salmon, *Salmo salar*, [35]. In addition, dietary proteins are closely linked to hepatic glucose content, since protein catabolism provides substrates that channel into gluconeogenic pathways [36]. In anurans, most of the research focused on nutrient biochemical pathways have been related to glucose mobilization from glycogen in liver and/or muscle for freezing adaptation during hibernation [37,38], glucose-lactate rates and changes on work output [39] and energy storage during dormancy [40]. None of these studies took into account either the dietary supply of the macronutrients or their relative ratio(s) and/or the effect(s) of diet composition on the dynamics of metabolism and energy production/expenditure.

Thus far, the question regarding to what extent anurans behave like strict carnivores in terms of nutrient utilisation and metabolism certainly remains unanswered. Therefore, the objective of this study was to evaluate the effect of different dietary macronutrient profiles on anuran digestion and metabolism at differing intake levels. For this purpose, we used as a model the African clawed frog, *Xenopus laevis*, an aquatic species widely utilised in biological, molecular and biochemical research [41]. We hypothesized that fat-rich diets, together with low intake levels, might inhibit the citric acid cycle, stimulating lipogenesis and hence influencing overall energy metabolism in adult *X*. *laevis*.

## Methods

### Animals and housing

The six-week study was conducted in the Laboratory of Animal Nutrition, following the guidelines of the EU Directive 2010/63/EU for animal experiments, and approved by the Ethical Committee for Use of Laboratory animals, from the Faculty of Veterinary Medicine and the Faculty of Bioscience Engineering of Ghent University, No. EC 2015/133. A total of 24 adult *Xenopus laevis* from an existing colony were randomly allocated by same-sex pairs into twelve 65 L aquaria (60×30×36 cm). This species has a marked sexual dimorphism; sexually mature males are typically 10–30% smaller than the females [42].

Tanks were equipped with a continuous supply of filtered and well-aerated tap water that had been previously dechlorinated; each aquarium was darkened on three sides with black plastic, and provisioned with PVC pipes as hiding sites. Photoperiod was 12:12 h light:dark, room and water temperature were 21.6°C and 20.1°C respectively. Average water quality parameters were: 366.7 mgL^-1^ hardness (Sera GmbH& Co, Germany), pH 7.5 (Merck KGaA, Darmstadt, Germany), <0.5 mg L^-1^ ammonia (Colombo, The Netherlands), 0.01–0.05 mgL^-1^ nitrites, 0.5–10 mgL^-1^ nitrates and 0.05 mgL^-1^ ammonium (JBL GmbH & Co., Germany). UV light was supplied by placing a UV bulb (Exo Terra 11W, Rolf C. Hagen Inc., Montreal QC, Canada) at 5 cm from the water surface twice a week on each tank, for an eight-hour exposure period.

### Experimental procedures

#### Digestibility trial

Aquaria (containing two same-sex animals with similar weights each) were randomly assigned to one or two diets treatments, six replicates per treatment. Each treatment consisted of an isoenergetic pelleted diet, made in the Laboratory of Animal Nutrition, especially formulated with two different carbohydrate:fat ratios, either 4.5:1 or 2.1:1, based in ranges reported for bullfrogs (*R*. *catesbeiana*) [17,21,22]. These ratios were established in order to change the dietary glucogenic (GLUC) and lipogenic (LIPO) proportion, and keeping the protein content stable to maintain a constant concentration of amino acids (Table 1). Before the trial, frogs were fed a maintenance diet (pellets, made with the same ingredients used in the treatment diets), and average food intake was calculated as a baseline for this study.

**Table 1:** Formulas and nutritional composition of diets offered to *X*. *laevis*.

Treatment	GLUCOGENIC DIET (GLUC)	LIPOGENIC DIET (LIPO)
**Macronutrients ratios**		
Carbohydrate:lipid ratio	4.5:1	2.1:1
Protein+carbohydrate:lipid ratio	9.0:1	4.7:1
**Ingredients (%)**		
Shrimp meal	57.70	57.70
Soybean meal	26.12	26.12
Wheat meal	13.80	-
Beef fat	0.22	5.22
Celite	-	8.8
Monocalcium phosphate	0.43	0.43
Premix^1^	0.43	0.43
Rice syrup^2^	1.29	1.29
**Proximate composition** **(dry matter basis)**		
Dry matter (%, as is basis)	87.9	89.0
Crude protein (%)	38.1	36.2
Fat (%)	8.6	13.5
Ash (%)	14.3	22.2
Carbohydrates^3^ (%)	39.0	28.1
Gross Energy^4^ (MJ/kg)	17.7	17.7
**Minerals (dry matter basis)**		
Calcium (%)	4.3	4.4
Phosphorus (%)	1.0	1.0
Magnesium (%)	0.2	0.2
Potassium (%)	1.4	1.1
Sodium (%)	0.4	0.4
Iron (mg/kg)	154.0	239.0
Zinc (mg/kg)	58.6	65.7
Manganese (mg/kg)	29.0	32.5
Copper (mg/kg)	42.5	36.5
Selenium (μg/kg)	527.0	527.0
**Amino acids** **(dry matter basis, g/kg)**		
Alanine	24.2	23.7
Arginine	25.6	24.6
Aspartic acid	41.1	40.0
Cysteine	5.1	4.7
Glutamic acid	59.5	54.6
Glycine	19.9	19.2
Histidine	10.5	9.6
Isoleucine	18.0	17.2
Leucine	30.5	29.0
Lysine	22.3	21.7
Methionine	7.8	7.4
Phenylalanine	18.8	18.0
Proline	19.1	17.3
Serine	20.4	19.2
Threonine	17.6	17.1
Tyrosine	13.3	13.0
Valine	20.8	19.7

^1^ ExoTerra Multivitamin supplement: 4.5% Ca, 0.00275% NaCl, 0.0033% K, 0.011% S, 2 mg/kg Mg, 77 mg/kg Fe, 2.5 mg/kg Cu, 6.5 mg/kg Zn, 0.75 mg/kg I, 6.5 mg/kg Mn, 2 mg/kg vitamin K, 0.009 mg/kg Biotin, 9 mg/kg Beta carotene, 22 mg/kg vitamin D_3_, 100 IU/kg vitamin E.

^2^ Used as binder of the mixture.

^3^ Carbohydrate = 100-Crude Protein-Fat-Ash.

^4^ Calculated.

The new diets were offered over 10 days prior to the experimental period, to acclimate frogs to the new food and to “wash out” the former diet from the digestive tract. This time was based on previous studies reporting a passage rate between 1–8 days in several frog species, *X*. *laevis* (unpublished data), *Cyclorana alboguttata* [43] and *Rana perezi* [44]. Once the trial began, frogs were fed three times per week (at 10:00 h) and daily intakes were determined by removing, drying and weighing the uneaten food from each aquarium after 40 min [45]. Animals were weighed at the beginning of the trial using a digital scale (OHaus CS Series ±1 g), and food intake (FI) per metabolic body weight (MBW) (g/(BW in kg)^0.75^) was calculated taking the average values of the animals from each aquarium.

Faeces were collected daily (when available) using a fish pipette of 30 ml, placing the sample in a 100 μm sieve mesh for water filtration, and then stored frozen at -20°C. At the end of the trial, faecal samples were pooled for all treatment groups and stored until analysis. Diets as well as faecal samples were analysed for proximate composition [46]. Apparent digestibility (aD) coefficients of dry matter (DM), crude protein (CP), fat and ash were calculated as follows:

$$\frac{aD\left(\%\right)=Nutrient\;intake–Nutrient\;in\;faeces\;X\;100}{Nutrient\;intake}$$

Amino acid content after defatting [47], and mineral profiles [48], were determined only in the food.

#### Blood sampling and analysis

At the end of the trial, animals were fasted for 24 h, weighed and anaesthetised using a solution of isofluorane mixed with distilled water and ultrasound gel, applied topically at a dose of 0.03 ml/g body weight [49]. Blood samples (max. 3% body weight) were drawn by heart puncture using a 1 mL syringe and a 26 G needle, placed onto Protein Saver cards (Whatman 903^TM^, GE Healthcare, Bio-Sciences Corp., MA, USA), and stored frozen at -20°C until analysis. After sampling, animals were recovered by spraying distilled water on their bodies and remained under observation until total recovery for ±5 h, then placed back into their respective aquaria. Acylcarnitines and amino acid profiles of dried blood spots were determined using tandem mass spectrometry [50] at the Laboratory for Metabolic Diseases at Ghent University Hospital. This technique has been widely applied to identify metabolic disorders biochemically detectable in human beings (adults and newborns) in both plasma and whole blood samples [51], and recently has been also employed in studies with animals [45, 52].

### Statistical analysis

The study design was completely randomized. All data are expressed as means and standard deviations (SD). Univariate ANOVA analysis was performed with treatment as fixed factor, food intake per metabolic body weight (FI/MBW) as covariable, and the interaction between Treatment X FI/MBW, to evaluate the effect on macronutrient digestibility as well as the amino acid and acylcarnitine profiles. In addition, regression analysis was performed to determine the relationship of macronutrient digestibility on FI/MBW. Statistical significance was accepted at p<0.05. Analysis was conducted using SPSS version 24.

## Results

**Fig 1:**
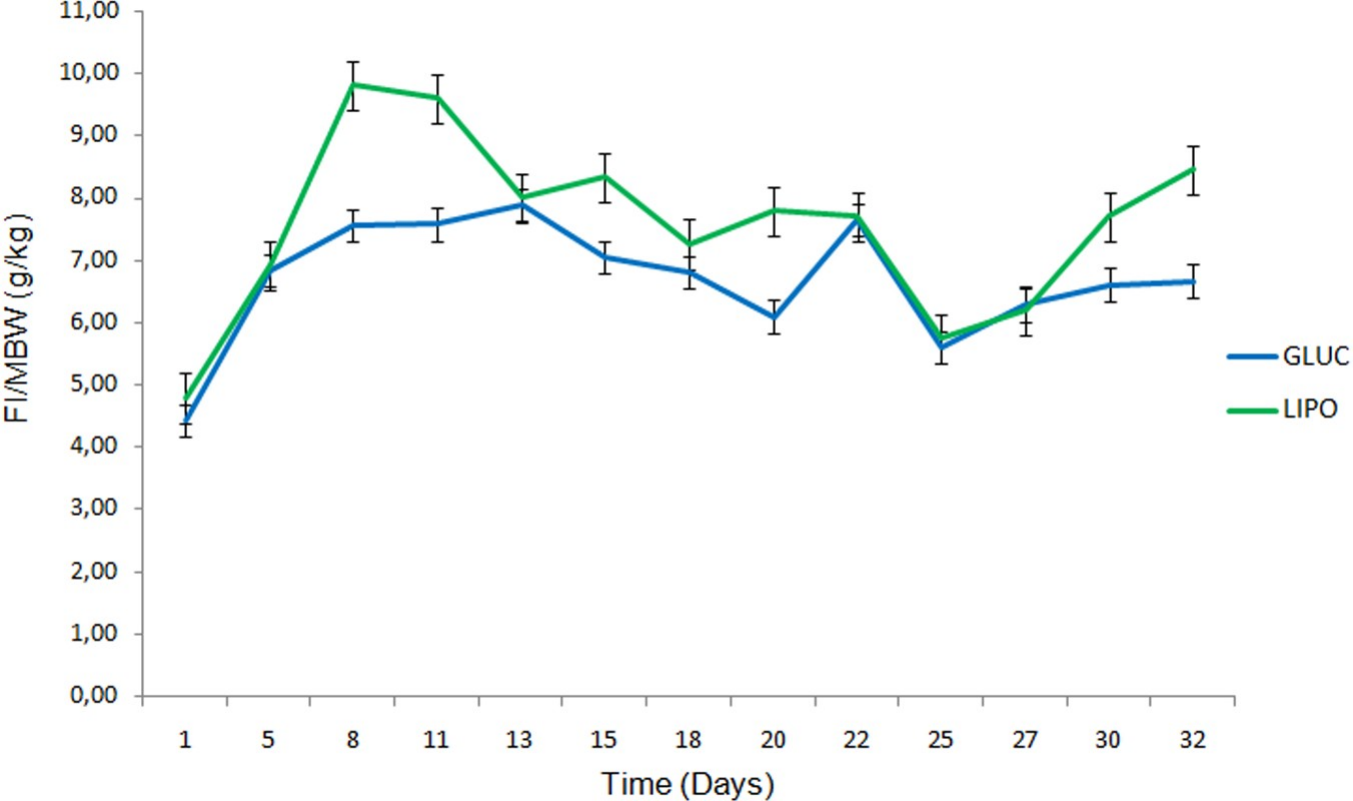
Food intake of isoenergetic glucogenic (GLUC) and lipogenic (LIPO) diets offered to adult *Xenopus laevis*. FI/MBW: Food intake per metabolic body weight.

**Fig 2:**
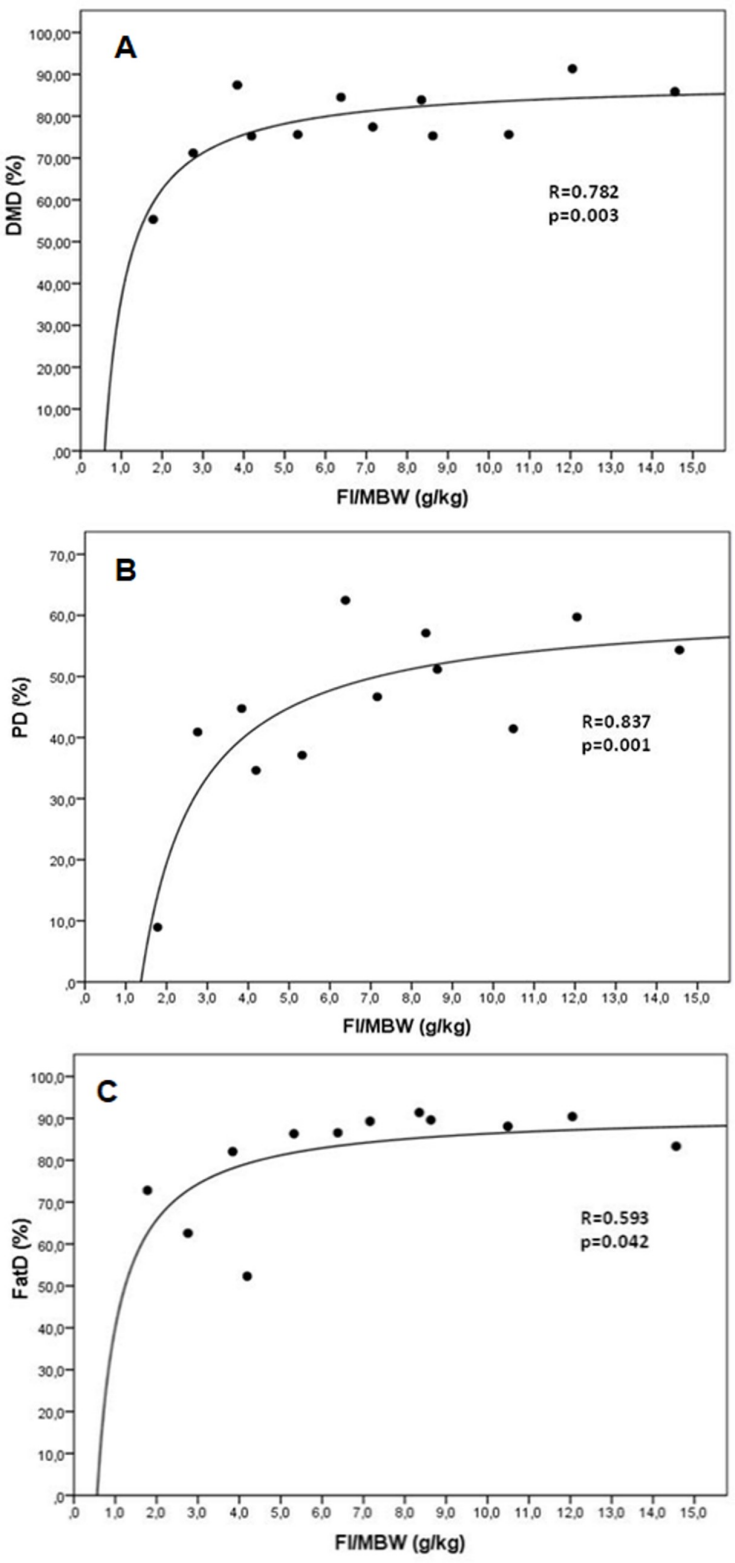
Regression analysis between the apparent digestibility of various macronutrients and food intake per metabolic body weight (FI/MBW) in *X*. *laevis*. A: DMD: dry matter digestibility, B: PD: protein digestibility, C: FatD: fat digestibility.

Average initial and final body weights were 68±33 and 67±34 g for animals assigned to GLUC, and 72±29 and 70±34 g for those assigned to LIPO diet treatments respectively. Weights did not vary (p = 0.894) over the 6-week trial period, nor they differ between treatment groups (p = 0.673). Food intake averaged 1.0±0.8 and 1.1±0.7 g total, or 6.7±4.8 and 7.6±3.1 g/kg MBW, for GLUC and LIPO diets, respectively. Although not significant (p>0.05), animals from LIPO presented an increase in food intake between days 5 and 13 of the study period; after 2 weeks, animals fed both treatments displayed a stable pattern of intake between 6.0 and 8.0 g/kg MBW until the end of the trial (Fig 1). Apparent digestibility coefficients of macronutrients and dry matter were numerically higher in LIPO diets, but significant differences (p<0.05) were obtained only in crude protein and fat digestibility, as an effect of FI/MBW (Table 2). Moreover, regression analysis indicated significant relationships (p<0.05) between apparent digestibility of dry matter and macronutrients (protein and fat) with food intake, where the positive association observed indicated how food intake might explain some of the variation seen in diet digestibility (Fig 2).

**Table 2:** Apparent digestibility of isoenergetic glucogenic (GLUC) and lipogenic (LIPO) diets offered to adult *X*. *laevis* at varying food intakes.

Apparent	GLUC	LIPO	*P* value		
Digestibility (%)			Treatment	FI/MBW	Treatment X FI/MBW
Dry matter	75.9±11.5	80.6±7.3	0.942	0.066	0.684
Crude Protein	36.9±15.3	53.0±8.3	0.196	0.039*	0.732
Fat	77.5±13.4	85.0±11.1	0.673	0.049*	0.377
Ash	-184.9±79.6	-149.1±46.5	0.358	0.161	0.589

FI = Food intake, MBW = Metabolic body weight. Treatment X FI/MBW: Interaction.

*Statistically significant (p<0.05)

Results from Table 3 show, in general, higher levels of blood metabolites in frogs fed diets containing more fat. Regarding amino acids and acylcarnitines, diet treatment had a significant effect only on the levels of malonyl (C3-DC), whereas FI/MBW had an important effect on methionine as well as several acylcarnitines tightly related to glucogenic amino acids and fatty acid synthesis. The interaction between treatment and FI/MBW also showed significant differences in malonyl, isovaleryl and hydroxyisovaleryl concentrations. This interaction was confirmed by the significant effect of FI/MBW on fat digestibility (Table 2). When metabolite ratios were evaluated, both treatment and FI/MBW, as well as the interaction between the parameters, had important effects on malonyl concentration related to total acylcarnitines.

**Table 3:** Selected blood amino acid and acylcarnitine profiles in adult *X*. *laevis* fed isoenergetic glucogenic (GLUC) or lipogenic (LIPO) diets at varying food intakes.

Metabolite	GLUC	LIPO		*P* value	
(μmol/L)			Treatment	FI/MBW	Treatment X FI/MBW
Amino acids					
Leucine	107.89±31.20	109.75±31.14	0.174	0.423	0.131
Methionine	14.24±4.04	17.55±4.18	0.599	0.005*	0.839
Valine	164.21±34.53	171.49±42.52	0.182	0.332	0.118
Carnitine esters					
Acetyl (C2)	3.69±0.91	4.80±1.09	0.707	0.158	0.533
Malonyl (C3-DC)	0.04±0.02	0.06±0.04	0.005*	0.033*	0.008*
3OH-Butyryl (3OH-C4)	0.09±0.05	0.12±0.05	0.313	0.805	0.134
3OH-Isovaleryl (3OH-C5)	0.42±0.14	0.70±0.20	0.136	0.006*	0.033*
Total LCFA	0.38±0.19	0.38±0.11	0.885	0.868	0.864
Total 3OH-LCFA	0.61±0.11	0.93±0.17	0.129	0.010*	0.031*
Ratios					
Met:CO	1.34±0.69	1.46±0.49	0.273	0.031*	0.256
C3-DC:Val	0.0002±0.0001	0.0004±0.0001	0.003*	0.013*	0.005*
3OH-C4:C2	0.03±0.01	0.03±0.01	0.072	0.187	0.054
C3-DC:Total Carnitine	0.002±0.001	0.003±0.002	0.001*	0.022*	0.001*
Tot. 3OH-LCFA:Total LCFA	1.85±0.64	2.62±0.99	0.140	0.019*	0.055

FI: Food intake, MBW: Metabolic body weight. Treatment X FI/MBW: Interaction, C: number of Carbons, 3OH: 3-hydroxy, DC: dicarboxylic acid in the acyl group, LCFA: long chain fatty acid, 3OH-LCFA: 3-hydroxy long chain fatty acid, Val: Valine, Leu: Leucine, Met: Methionine.

*Statistically significant (p<0.05)

**Fig 3:**
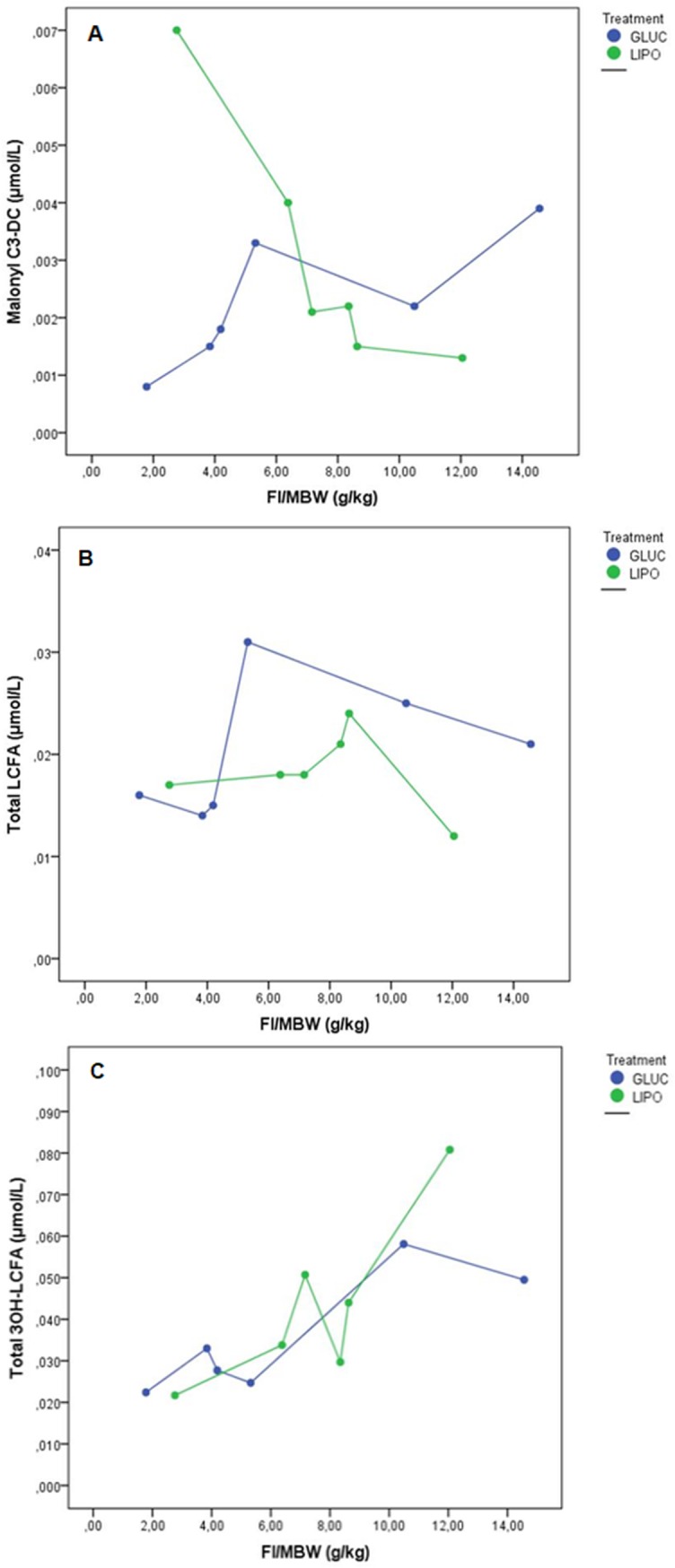
Metabolites related to fatty acids metabolism in adult *Xenopus laevis* fed isoenergetic glucogenic (GLUC) or lipogenic (LIPO) diets. FI: Food intake, MBW: metabolic body weight, A: malonyl-CoA, B: LCFA: long chain fatty acids, C: 3OH-LCFA: 3 hydroxy long chain fatty acids.

**Fig 4:**
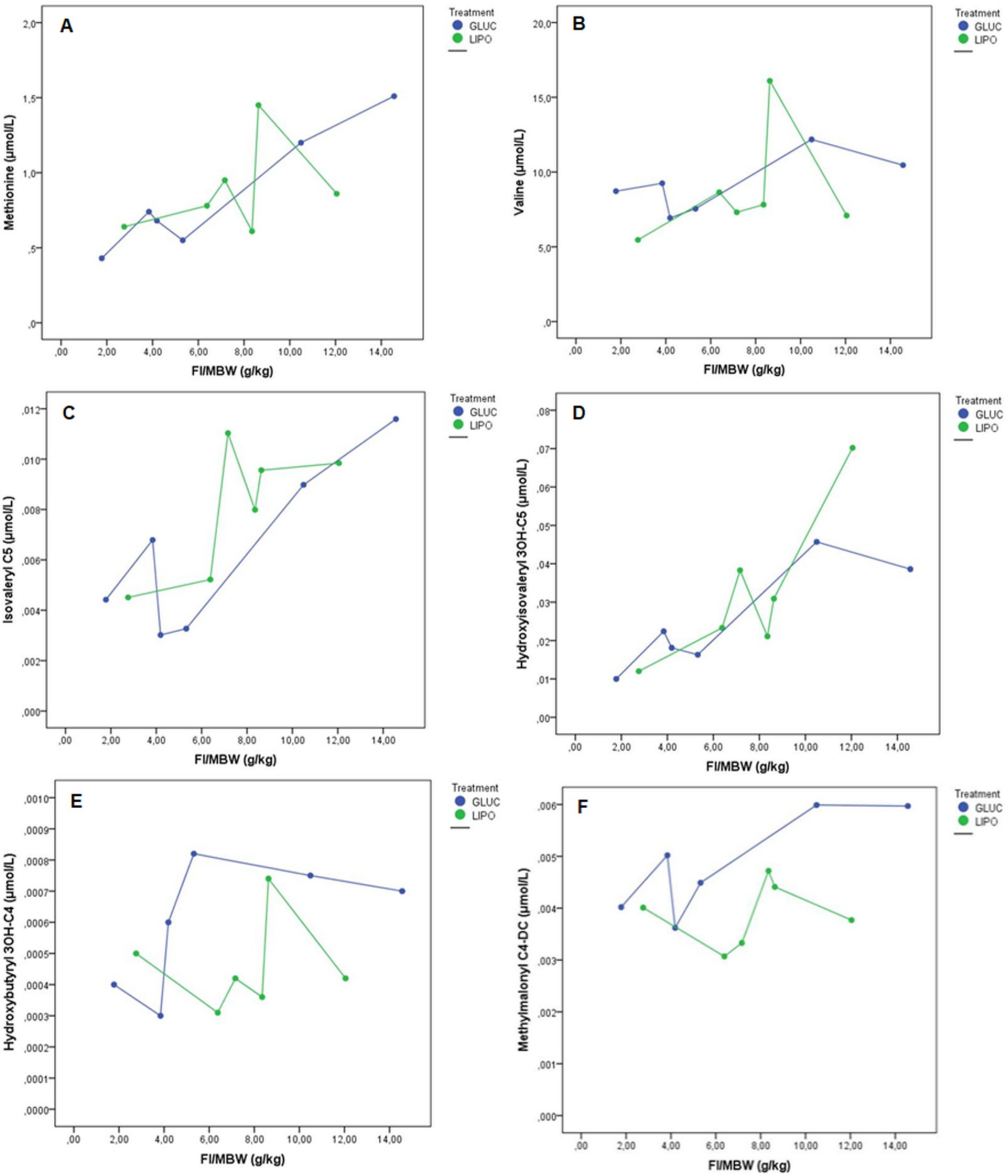
Metabolites related to amino acid catabolism in adult *Xenopus laevis* fed isoenergetic glucogenic (GLUC) or lipogenic (LIPO) diets. FI: Food intake, MBW: metabolic body weight. A: Methionine, B: Valine, C: Isovaleryl, D: Hydroxyisovaleryl, E: Hydroxyisobutyryl, F: Methylmalonyl.

Metabolite concentrations analysed in relation to food intake showed an inverse response of malonyl carnitine to diets, decreasing with the lipogenic and increasing with the glucogenic diet (Fig 3A). Likewise, long chain fatty acids (LCFA) diminished while 3OH-LCFA rose with both diets (Fig 3B and 3C). Compounds related to amino acid metabolism are shown in Fig 4. Methionine and valine (4A, 4B) as well as other metabolites related to amino acid catabolism (4C, 4D, 4E, 4F), presented an increase with food intake in the glucogenic diet, whereas the response of the lipogenic diet was quite variable.

## Discussion

In the present study, independent of diet composition, food intake drove most of the changes in the metabolism of *X*. *laevis*. Food intake in animals is controlled by the hypothalamus, regulated by several satiety-related gut hormones, in association with other factors [6]. The increase in food intake during the first days of the trial, followed by a later plateau (Fig 1), might be a consequence of self-regulation mechanisms as a result of a positive energy balance following intake of high carbohydrate/fat diets, where leptin concentrations increase and suppress neuropeptide Y. This self-regulation is already reported in several diverse species, including ponies [53], rats [54] and dogs [55], among others.

The apparent digestibility coefficients obtained in *X*. *laevis* presented variations when compared to studies performed in the bullfrog *R*. *catesbeiana*. Higher dry matter digestibility values (82.5 and 95.4%) were already reported in frogs fed diets containing fish meal [15,17]. On the other hand, protein digestibility in this study was relatively low, possibly due to the ingredients utilised in the diets. Values obtained from the lipogenic diet agreed with diets including feather (55.1%), and sardine meal (54.4%) [16]. Conversely, shrimp meal (the major ingredient of the diets in the present study) has shown 65% protein digestibility *in vitro*, as well as 22% chitin digestibility [56]; ingredient composition may have resulted in the low coefficients obtained through poor digestibility of the shrimp exoskeleton constituent [57]. Fat digestibility agreed with previously-reported values for *R*. *catesbeiana* frogs fed diets based on poultry by-products, meat and bone, as well as soybean meals [16].

Macronutrient digestibility coefficients showed no differences in regard to diets consumed, although they were numerically higher in frogs fed the lipogenic diet indicating that, at least, most of the dry matter from both diets was well digested (except protein) [6]. However, there was a noteworthy effect of food intake in those values (Table 2), where a clear positive association was demonstrated in the beginning of the trial and stable digestibility values were seen after intakes of 5 g/kg MBW (Fig 2). Similar responses have been reported in dry matter and protein digestibility in Wistar rats [58] as well as rabbits of mixed breeds (New Zealand White X Chinchilla) [59]. Nonetheless, it must be acknowledged that both intake and digestibility are also tightly related to passage rate and the physical characteristics of food. Thus, an increase in intake usually leads to an increase in the passage rate of the digesta, causing a reduction in digestibility since ingesta is exposed to enzymatic activity for a shorter period of time [6]. These relationships between food intake and digestibility, as well as between food intake and passage rate, have been studied in detail in herbivores (ruminants and other foregut fermenters), and are recognized as major factors influencing nutrient utilisation and digestive efficiency [60,61]. Amphibians, on the other hand, have a low metabolic rate compared to endotherms [62], and several studies have shown that feeding and gut passage can vary at low temperatures [63] as well as during aestivation [43]. In the case of anurans not undergoing aestivation or hibernation processes, however, the point at which food intake begins to negatively affect digestibility of the diets consumed is not yet known.

Free amino acid and acylcarnitine concentrations were in general higher in animals fed the lipogenic diet, with food intake having a predominant influence on the responses of the metabolites. Some interactions between food intake and diet composition were detected, revealing a co-dependence in the use of substrates for fatty acid metabolism related to fat intake and digestion together with the level of dietary fat. From the fatty acid metabolism side, malonyl carnitine presented inverse responses to the diets when evaluated in terms of intake, with increasing values from animals fed the glucogenic diet (Fig 3A). This implies that there is an excess of substrate (acetyl-CoA) over metabolic demands, stimulating therefore fatty acid synthesis through the glycolytic pathway, which is activated when there is a surplus of glucose and consequently of pyruvate. When this occurs, acetyl-CoA is mobilised from the mitochondria to the cytosol via the citrate shuttle, involving the carboxylation of acetyl-CoA to form malonyl-CoA and triggering the lipogenic cascade [26,64]. Furthermore, waning in overall LCFA with the subsequent incremental increases in their activated form(s), the 3OH-LCFA (Fig 3B and 3C), together with the significant effect of the lipogenic diet, indicates that, in turn, dietary fat is used to yield acetyl-CoA through ß-oxidation. The rate of this process is a function of plasma concentrations of these substrates, switching off the fatty acid synthesis by inhibiting the malonyl-CoA concentrations, all regulated by the action of the hormones insulin and glucagon [29,65].

On the other hand, amino acids and their catabolites also conferred further information on macronutrient utilisation in frog metabolism (Fig 4). Methionine on average was higher in the lipogenic diet, but its variation related to food intake showed a pattern of increase only in the glucogenic diet, while the levels tended to go down with the increase of food intake in the lipogenic diet. In this case, in animals fed on the lipogenic diet, methionine could have been directed to the synthesis of carnitine, activating the carnitine/fatty acid shuttle, hence leading to ß-oxidation in order to utilise the higher abundance of dietary fat as an energy source [29,66]. Moreover, isovaleryl-CoA is one of the esters obtained from the catabolism of leucine, the only pure ketogenic amino acid. Its response indicates that, irrespective of diet, the higher the food intake, the more leucine is utilised to cleave the isovaleryl-CoA in the liver to yield acetyl-CoA and acetoacetate by other routes. Together with isovaleryl carnitine, the rise of 3-hydroxyvaleryl carnitine suggests that likely there is an excess of leucine that is no longer necessary as a ketogenic energy source, deviating therefore its catabolism to the production of this end compound [26]. The catabolism of the amino acids isoleucine and valine is illustrated with the responses obtained in the concentrations of 3-hydroxybutyryl and methylmalonyl carnitines. Both metabolites showed an increase as food intake increased, though this pattern was notable in the glucogenic diet before the intake of 8 g/kg MBW of food, suggesting that, despite high dietary carbohydrate supply, frogs prefer to catabolise amino acids to render propionyl CoA and/or methylmalonyl CoA that can enter the citric acid cycle through succinyl-CoA instead of acetyl-CoA. This metabolic behaviour resembles that of strict carnivores, where protein and fat are the main sources of energy. Conversely, the 3-hydroxybutyryl values also suggest that, at low intakes, frogs do not seem to turn to ketone production, indicating a possible down-regulation of metabolism instead when intake is low [26,36,52,66], which seems logical given the physiological capacity of regulation of metabolic rates as an ecological adaptation of ectothermic animals [61].

In conclusion, the present study revealed that, regardless of diet composition, there is a remarkable impact of food intake on macronutrient digestibility as well as several circulating amino acids and acylcarnitine metabolites in this frog model. Although physicochemical properties of ingredients might influence such responses, this study provides evidence that various parameters are involved in the metabolic reaction to diets, which is highly related to feeding strategy. Acylcarnitine profiles were shown to be a valuable technique to evaluate such responses. Metabolite levels reflected the low abilities of this species to use carbohydrates as energy sources, supporting the theory that—at least regarding metabolites available for the citric acid cycle—frogs resemble strict carnivores more so than omnivores. As an amphibian model for nutrition research, *Xenopus laevis* showed engaging insights on systemic regulation of nutritional metabolism that previously were lacking in this animal group, reinforcing the need for further investigation into metabolism at the biochemical level in anurans.
